# Discrimination by X-ray fluorescence analysis of elemental concentrations in healthy and diseased rat tissues

**DOI:** 10.1080/07853890.2021.1896894

**Published:** 2021-09-28

**Authors:** Luísa Zagalo, Gonçalo Pereira, Pedro Oliveira, Maria João Oliveira, Luísa Gonçalves, Carlos Zagalo, José Brito

**Affiliations:** a Centro de Investigação Interdisciplinar Egas Moniz (CiiEM), Egas Moniz Cooperativa de Ensino Superior, Caparica, Portugal; b Instituto de Ciências Biomédicas Abel Salazar, Porto, Portugal

## Abstract

**Introduction:**

Elements such as Ca and P are involved in important physiological processes and their concentrations may differ significantly between healthy and diseased individuals [1]. Investigation of the role of single elements has been questioned because it ignores important interactions amongst various elements [2]. Often, when concentrations of various elements are obtained in the same study, comparisons between healthy and diseased tissues, or correlations between those elements, both intrinsically multivariate, are implemented with univariate methods, which may result in inflated or unobserved existing effects [3,4]. The methodologies in here complement multielement determinations by X-ray fluorescence spectroscopy (XRF) with multivariate data analysis methodologies, and offer an important contribute to fill existing gaps in current knowledge of the role elements in such metabolic pathways.

**Materials and methods:**

Here, the XRF technique is applied to assess the concentration profile of Ca and P, in samples of 4 groups of Wistar rat tibiae, aiming at assessing the musculoskeletal impact of the synergy effects of an environmental factor and a disease with recognised systemic effects. Each group contained 12 animals: exposed to low-frequency noise (yes/no) and diabetic/hyperglycaemic rats (yes/no). Animals from each group were randomly divided into three timepoints and sacrificed after 1, 6 and 12 weeks of exposure. In view of this experimental design, Two-Way ANOVA has been used to assess the effect of main factors and interaction of those factors on the dependent variables, after validation of model assumptions.

**Results:**

For bone Ca concentration, there was no interaction between the two main factors, with no effect due to noise exposure (*p* = .501) but with a significant effect due to diabetes (*p* = .038), with observed power of 68%. A similar outcome was observed for bone P concentration (*p* = .456), with no interaction between factors, no effect due to noise, but with significant impact due to diabetes (*p* = .003), with observed power of 69.2%. In both cases, the concentrations of Ca and P were decreased in the group of diabetic animals. For the ratio Ca/P no significant effects or interactions were detected (*p* = .140).

**Discussion and conclusions:**

XRF spectrometry is clearly a highly promising technique to be used in the discrimination of elemental concentrations in healthy and diseased rat tissues. As this is a pioneer investigative work and the number of animals included is limited in this first approach, it is early to anticipate definite results. However, we can, in the meantime, observe the presence of diabetic-induced osteopenia, with no effects resulting from exposure to noise.
